# The effect of the heart rate lowering drug Ivabradine on hemodynamics in atherosclerotic mice

**DOI:** 10.1038/s41598-018-32458-3

**Published:** 2018-09-18

**Authors:** R. Xing, A. M. Moerman, R. Y. Ridwan, K. van Gaalen, E. J. Meester, A. F. W. van der Steen, P. C. Evans, F. J. H. Gijsen, K. Van der Heiden

**Affiliations:** 1 Department of Biomedical Engineering, Thorax center, Erasmus University Medical Center, Rotterdam, The Netherlands; 2 Department of Molecular Genetics, Erasmus University Medical Center, Rotterdam, The Netherlands; 3 Department of Radiology & Nuclear Medicine, Erasmus University Medical Center, Rotterdam, The Netherlands; 4 Department of Infection, Immunity and Cardiovascular Disease, University of Sheffield, Sheffield, United Kingdom

## Abstract

The heart rate lowering drug Ivabradine was shown to improve cardiac outcome in patients with previous heart failure. However, in patients without heart failure, no beneficial effect of Ivabradine was observed. Animal studies suggested a preventive effect of Ivabradine on atherosclerosis which was due to an increase in wall shear stress (WSS), the blood flow-induced frictional force exerted on the endothelium, triggering anti-inflammatory responses. However, data on the effect of Ivabradine on WSS is sparse. We aim to study the effect of Ivabradine on (i) the 3D WSS distribution over a growing plaque and (ii) plaque composition. We induced atherosclerosis in ApoE^−/−^ mice by placing a tapered cast around the right common carotid artery (RCCA). Five weeks after cast placement, Ivabradine was administered via drinking water (15 mg/kg/day) for 2 weeks, after which the RCCA was excised for histology analyses. Before and after Ivabradine treatment, animals were imaged with Doppler Ultrasound to measure blood velocity. Vessel geometry was obtained using contrast-enhanced micro-CT. Time-averaged WSS during systole, diastole and peak WSS was subsequently computed. Ivabradine significantly decreased heart rate (459 ± 28 bpm vs. 567 ± 32 bpm, p < 0.001). Normalized peak flow significantly increased in the Ivabradine group (124.2% ± 40.5% vs. 87.3% ± 25.4%, p < 0.05), reflected by an increased normalized WSS level during systole (110.7% ± 18.4% vs. 75.4% ± 24.6%, p < 0.05). However, plaque size or composition including plaque area, relative necrotic core area and macrophage content were not altered in mice treated with Ivabradine compared to controls. We conclude that increased WSS in response to Ivabradine treatment did not affect plaque progression in a murine model.

## Introduction

Atherosclerosis is characterized by the accumulation of lipids, fibrous tissues and inflammatory cells in the arterial wall. The initiation of atherosclerosis is regulated by low wall shear stress (WSS), while high WSS is known to be athero-protective in the early phase of the disease^1,2^. When the disease progresses, the plaques can grow to the extent that they affect lumen diameter, resulting in changes in WSS over the plaque^3–5^. How these changes in WSS affect progression of the disease is still a topic under investigation^3,4^.

Increased heart rate has been established as a risk factor for cardiovascular disease in a healthy population and in a population with a history of various heart diseases^6–8^. The heart rate lowering drug Ivabradine acts on the cardiac pacemaker I_f_ ion current which is highly expressed in the sinoatrial node. By inhibiting the I_f_ current, the diastolic depolarization phase is delayed, thus the heart rate is selectively reduced^9^. Several clinical studies showed that Ivabradine improved cardiac outcome in patients who had coronary artery disease with previous heart failure^8,10,11^. However, a recent study suggested no beneficial effect of Ivabradine on patients who had coronary artery disease without heart failure^12^. Since the reported results are event-based only, size and composition of these coronary artery plaques is unknown, and hence the effect of Ivabradine on plaque progression is not conclusive.

Experimental evidence of lowering heart rate on reduced plaque burden was previously demonstrated by Beere *et al*.^13,14^. Animal studies have shown that Ivabradine reduced heart rate and decreased oxidative stress, prevented endothelial dysfunction and thus reduced atherosclerosis^15–20^. These studies suggested that the effect of Ivabradine was exerted via alteration of the hemodynamic environment. However, the mechanism of the beneficial effect of Ivabradine on hemodynamics has not been established. We and others suggested that by increasing the duration of the diastolic phase and thus lowering the heart rate, Ivabradine indirectly alters the local hemodynamic environment^16,21^. In our previous study, using LDLR^−/−^ mice, an increased WSS in the aortic arch was observed upon treatment with Ivabradine^21^. WSS was calculated using averaged flow measurement from multiple mice and using the geometry of one mouse. Also, the effect of Ivabradine on plaque progression and/or composition was not investigated. Most animal studies focused on the **preventive** effect of Ivabradine: the animals were treated with Ivabradine simultaneously with the onset of high fat diet. This does not reflect the clinical situation where patients with known cardiovascular disease showed improved secondary outcomes after Ivabradine treatment^8,10,11^. In other studies, endothelial dysfunction was first induced by 4–6 weeks of high fat diet and then Ivabradine was administered^17,21^. However, this duration of high fat diet was too short to induce formation of an advanced plaque. Therefore, a **curative** study design where Ivabradine treatment is administered after the formation of an advanced plaque is more clinically relevant.

Therefore, in this study, we performed a detailed analysis of the effect of Ivabradine on WSS and atherosclerotic plaque progression and composition in a mouse model of atherosclerosis. We used the well-established cast mouse model on ApoE^−/−^ mice, in which a plaque with characteristics of a vulnerable phenotype is induced upstream of the cast. After formation of this plaque, animals were treated with Ivabradine and its effect on blood flow parameters, WSS, and its potential effect on plaque composition was analyzed.

## Methods

To study wall shear stress (WSS) *in vivo*, we need to acquire parameters including mouse-specific vessel geometry, blood flow velocity and vessel diameter. The vessel geometry can be obtained using high-resolution micro-CT imaging, while blood flow velocity and vessel diameter can be assessed using ultrasound imaging. This information can then be processed and used to compute WSS.

### Animals, cast placement and Ivabradine treatment

Female ApoE^−/−^ mice on a C57BL/6 J genetic background (n = 18) were purchased from Charles River (Maastricht, The Netherlands). At 13 weeks of age, normal chow diet was replaced with an atherogenic high fat diet (Altromin Spezialfutter, Germany) and provided *ad libitum*. Cast surgery was performed two weeks later on the animals under isoflurane-induced anesthesia^5,22,23^. A constrictive cast with an internal diameter of 400 μm tapering to 200 μm over 1.5 mm was placed around the right common carotid artery (RCCA), leading to changes in local WSS environment and subsequent plaque development. Five weeks after the cast placement, mice (n = 9) were treated with Ivabradine in drinking water *ad libitum* for a duration of 2 weeks (162 mg/L, equivalent to 15 mg/kg/day) or with regular drinking water as control (n = 9). This optimal dose was determined in our previous publication^21^, where we demonstrated a biological effect of Ivabradine on the arterial wall and a reduction in heart rate by approximately 20%, proportional to that observed in Ivabradine-treated patients^10^.

The amount of drinking water was monitored for mice and refreshed twice a week. A pre-study power analysis was performed based on our previous experience with this dose of Ivabradine^21^, where the smallest significant biological effect of Ivabradine on the arterial wall was observed in the endothelial VCAM-1 expression in the outer curvature of the mouse aorta (reduction by 80% with a standard deviation of 45%). To reach a power of 80%, 5 experimental animals are required to obtain statistical significance (p < 0.05). An estimated 40% loss of experimental animals due to the multiple anesthesia moments was incorporated and ethical approval for 9 experimental animals per group was obtained. All animal experiments were performed conform to the guidelines from Directive 2010/63/EU of the European Parliament on the protection of animals used for scientific purposes, and were approved by the ethical committee of Erasmus MC Rotterdam.

### Animal Imaging Experiments

Doppler Ultrasound Imaging was performed using Vevo 2100 (VisualSonics). Blood velocity waveform upstream of the RCCA was measured with a 40-MHz transducer in the pulse-wave mode. The distinction between the systole and diastole of the cardiac cycle was determined by the dicrotic notch identified on the RCCA blood velocity waveforms^24^. RCCA diameter at systole or diastole was measured in the M-mode at the same location of the velocity measurement, allowing conversion of blood velocity (mm/s) to flow (mm^3^/s) assuming a parabolic velocity profile and a circular geometry of the vessel cross-section. The parameters were determined at 5 weeks after cast placement (t = 0, baseline) and 3 days after the treatment of Ivabradine (t = 3) in both control and Ivabradine-treated mice. Animals were anesthetized under isoflurane (1.5–3% with 1 L/min oxygen) during the measurement while heart rate was measured by ECG during 24–38 min after beginning anesthesia. The body temperature was maintained between 35.8 °C to 37.7 °C during the procedure by a heating pad and a heating lamp.

At t = 3, contrast-enhanced micro-CT imaging was performed using a Quantum FX (PerkinElmer). The scanning parameters used were 90kvp, 160 µA with 20 mm field of view and a scan duration of 4.5 min. Contrast agent eXIA 160 (Binitio Biomedical, Canada) was used with an injection dose of 150 µl/25 grams of body weight. Micro-CT imaging was performed directly before the ultrasound imaging. Images in Hounsfield unit (HU) were reconstructed with an isotropic resolution of 40 µm. However, 7 out of 18 mice died 1–2 days after the imaging point at t = 3. This premature death is probably due to the combination of repeated anesthesia and the use of imaging contrast agent. As a result, WSS simulation (n = 5 control and n = 5 Ivabradine-treated mice) and histology (n = 4 control and n = 5 Ivabradine-treated mice) analyses were performed in a subset of animals.

### RCCA lumen surface reconstruction and CFD simulation

RCCA lumen geometry was segmented using previously established protocols in MeVisLab (MeVisLab 2.2.1) and Matlab (Matlab R2017a)^25^. RCCA lumen surface was reconstructed from its origin at the bifurcation of the brachiocephalic artery, to its bifurcation into the internal and external carotid artery.

RCCA lumen surface was smoothed using the Vascular Modelling Tool Kit (VMTK 1.2). The superfluous ends at the proximal and distal side of the vessel surface were clipped and flow extensions were added. A volume mesh with prism layers was then generated using ICEM (ICEM-CFD 14.5, Ansys) and a mesh-independent solution with approximately 580,000 elements was obtained. The Surface area of the RCCA inlet was derived, enabling the calculation of location-specific blood flow velocity as an inlet boundary condition.

The Navier-Stokes equations were solved by computational fluid dynamics (CFD) using Fluent (Fluent 17.1, Ansys). Blood was modeled to be incompressible. A constant viscosity of 3.5 × 10^−3^ kg·m^−1^·s^−1^ was used, and vessel wall was assumed to be rigid. A parabolic velocity profile was imposed as inlet boundary condition. The time-dependent blood flow wave form was derived from Doppler velocity measurements. For the outlet boundary condition, zero pressure was used. Time-dependent flow simulations were performed for 2 cardiac cycles with 100 time steps per cycle. WSS was derived from the computed velocity field. Finally, post-processing and analysis were performed in CFD-Post (CFD-Post 14.5, Ansys) and Matlab to quantify time-averaged WSS (TAWSS), TAWSS during systole and diastole, and peak WSS.

### Histological and immunohistochemical analyses

RCCA vessel samples of Ivabradine-treated mice (n = 5) and control mice (n = 4) were harvested and embedded in paraffin for histological analysis. Serial sections of 5 µm thick were collected every 50 µm, from the brachiocephalic bifurcation proximally to the carotid bifurcation distally. The sections were stained with H&E for general plaque morphology, Resorcin-Fuchsin for collagen, CD31 for endothelium (1:20, Dianova, Germany) and CD68 for macrophages (1:100, Bio-Rad, USA). Plaque area and macrophage area were quantified (BioPix iQ3.2). Necrotic core was defined as a-cellular, a-nuclear areas, free of H&E staining, essentially as described previously^5^

### Statistical analysis

Data analysis was performed in Matlab (Matlab R2017a) and RStudio (RStudio 1.0.153). Data are presented as mean ± SD. The normality of the data was evaluated by Shapiro–Wilk test. If the data was normally distributed, the student t-test was used for further analyses. For data that was not normally distributed, a non-parametric Wilcoxon signed-rank test was used. Differences between the Ivabradine-treated group and the control group were evaluated using an unpaired, two-tailed students’ t-test or Wilcoxon signed-rank test. Differences between the 2 time points (t = 0 vs. t = 3) within the same group were evaluated using a paired, two-tailed students’ t-test or non-parametric Wilcoxon signed-rank test. The results were considered significant if the p-value < 0.05.

## Results

### Ivabradine reduced heart rate

**Figure 1:**
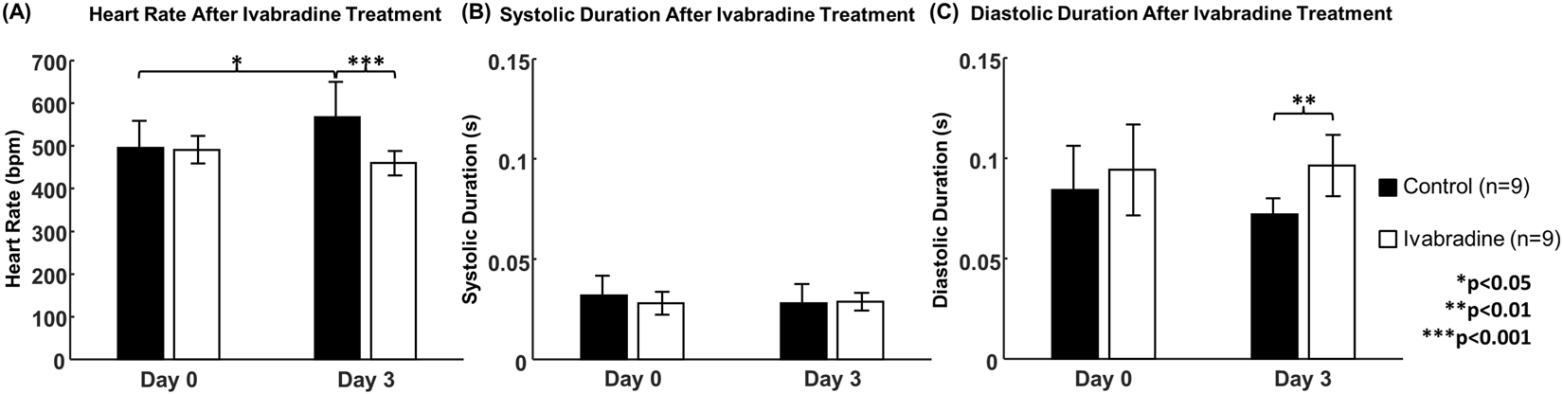
Differences of cardiac parameters between control mice (n = 9) and Ivabradine-treated mice (n = 9). The graphs are shown as mean ± standard deviation. (**A**) Heart rate; (**B**) Duration of the systolic phase; (**C**) Duration of the diastolic phase. Note: *p < 0.05, **p < 0.01, ***p < 0.001.

Heart rate at baseline (t = 0 days) and after administration of Ivabradine (t = 3 days) are shown in Fig. 1A. At baseline, there was no difference in heart rate between the control and the treatment group (495 ± 64 bpm vs. 491 ± 83 bpm, p > 0.05). After 3 days treatment, heart rate in mice treated with Ivabradine was 459 ± 28 bpm, which was significantly lower compared to that of the control group (567 ± 32 bpm, p < 0.001). Interestingly, heart rate in control mice increased significantly from baseline to 3 days treatment (p < 0.05). The normalized values are listed in Table 1. Compared to the control mice, heart rate of the Ivabradine-treated mice reduced 18.1%. The data for each individual mouse are presented in the Supplemental Fig. 1.

**Table 1:** Normalized Cardiac and Flow Parameters.

Parameter	Control (n = 9)	Ivabradine (n = 9)	P-value
Heart Rate	116.8% ± 22.1%	95.9% ± 17.2%	0.03*
Systolic Duration	89.5% ± 14.4%	106.4% ± 29.1%	0.14
Diastolic Duration	90.2% ± 22.5%	108.2% ± 31.0%	0.18
Average Blood Flow	90.3% ± 35.9%	125.8% ± 48.5%	0.10
Peak Blood Flow	87.3% ± 25.4%	124.2% ± 40.5%	0.03*

### Ivabradine prolonged the duration of the diastolic phase

The reduction of heart rate after the administration of Ivabradine resulted in a prolonged cardiac cycle compare to control mice (p < 0.001, data not shown). The effect of Ivabradine was further investigated by dividing the cardiac cycle into the systolic and the diastolic phase. As shown in Fig. 1B, Ivabradine did not alter the duration of the systolic phase (0.028 ± 0.010 s vs. 0.029 ± 0.004 s). However, the diastolic phase was significantly longer in mice treated with Ivabradine compared to that of the control group (Fig. 1C, 0.072 ± 0.008 s vs. 0.096 ± 0.015 s, p < 0.01). Table 1 shows the normalized cardiac duration of the systolic and the diastolic phase. The data for each individual mouse are presented in the Supplemental Fig. 2.

### Ivabradine did not alter blood flow in the RCCA

The effect of Ivabradine on the flow environment in the RCCA was studied. There were no differences in absolute time-averaged or peak blood flow between the Ivabradine-treated mice and the control mice. However, normalized peak flow was 124.2% ± 40.5% after the Ivabradine treatment, while it decreased to 87.3% ± 25.4% in the control group (p < 0.05). The data for each individual mouse are presented in the Supplemental Fig 3.

### Ivabradine affected wall shear stress in the RCCA during systole

**Figure 2:**
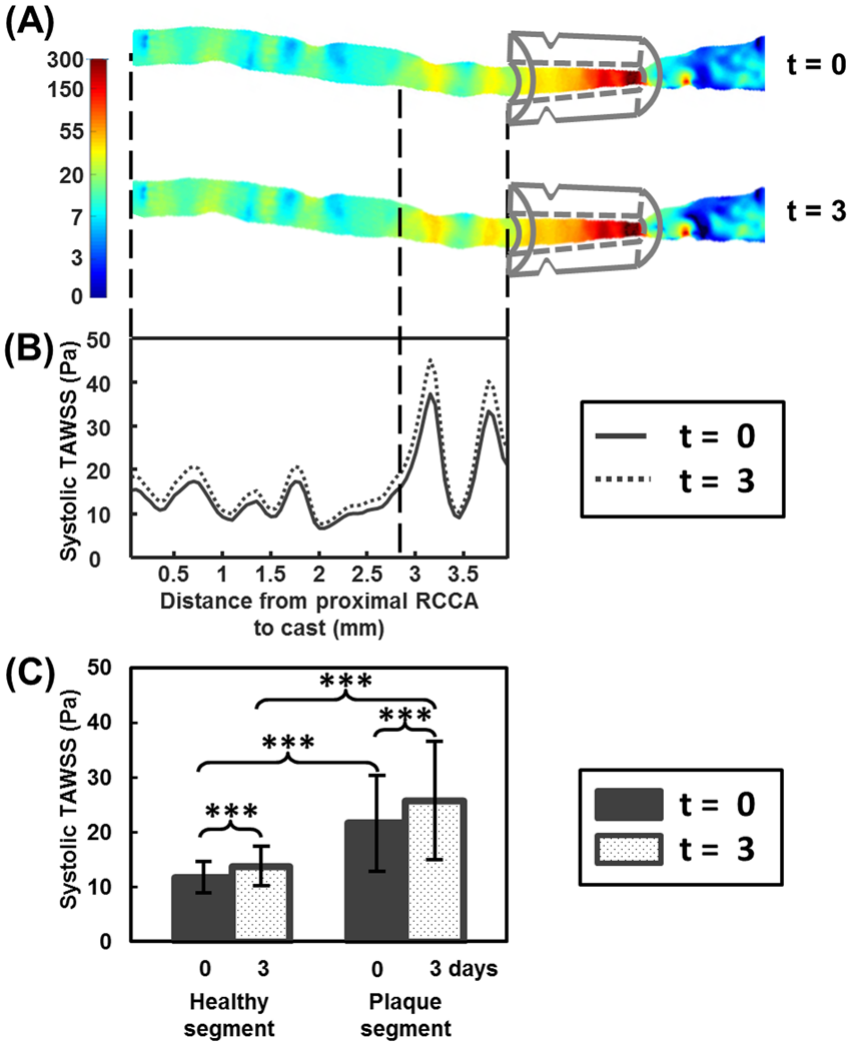
Ivabradine increased TAWSS during systole. (**A**) 3D distribution of TAWSS during systole in the RCCA before (t = 0) and after Ivabradine (t = 3) in a representative mouse; (**B**) Circumferentially-averaged WSS along the proximal RCCA; The proximal RCCA was divided into the healthy and the plaque segment, indicated by the vertical dashed line; (**C**) TAWSS during systole in both the healthy and the plaque segments were significantly increased after treatment with Ivabradine. Note: ***p < 0.001.

Different WSS parameters in the healthy segment and the plaque segment of the RCCA were investigated. After normalization, TAWSS during systole in the RCCA was significantly higher in animals treated with Ivabradine compared to the control mice (Table 2, p < 0.05). Figure 2 illustrates the distribution of TAWSS during systole in the RCCA of a representative mouse. Treatment of Ivabradine significantly increased TAWSS during systole in both the healthy segment and the plaque segment (Fig. 2C). Also, peak WSS increased significantly in the upstream RCCA after the treatment of Ivabradine (p < 0.05, Table 2). However, there were no significant differences between the control and the Ivabradine-treated mice in terms of the other WSS parameters investigated, including TAWSS or TAWSS during diastole. Table 2 summarizes the normalized WSS parameters. The data for each individual mouse are presented in the Supplemental Figs 4 and 5.

**Table 2:** Normalized WSS Parameters.

WSS Parameter	Control (n = 5)	Ivabradine (n = 5)	P-value
TAWSS Healthy Segment	77.9% ± 21.2%	99.7% ± 22.9%	0.16
TAWSS Plaque Segment	77.4% ± 21.4%	99.4% ± 23.4%	0.16
TAWSS Systole Healthy Segment	74.6% ± 21.5%	110.7% ± 18.2%	0.02*
TAWSS Systole Plaque Segment	74.1% ± 21.5%	110.7% ± 18.4%	0.02*
TAWSS Diastole Healthy Segment	80.3% ± 20.8%	95.6% ± 24.3%	0.32
TAWSS Diastole Plaque Segment	80.0% ± 21.1%	95.0% ± 24.6%	0.33
Peak WSS Healthy Segment	75.4% ± 22.9%	111.9% ± 22.1%	0.03*
Peak WSS Plaque Segment	74.3% ± 23.1%	111.6% ± 22.9%	0.03*

### Ivabradine did not change plaque composition

After 2 weeks of Ivabradine treatment, RCCA vessel samples were harvested for histological analyses. Plaque compositional parameters including plaque area, plaque length, relative necrotic core area and relative macrophage area were characterized. None of the parameters were significantly different between the control group and the Ivabradine-treated group (Table 3).

**Table 3:** Plaque Composition Parameters.

Plaque Composition Parameters	Control (n = 4)	Ivabradine (n = 5)	P-value
Plaque Area (mm^2^)	0.32 ± 0.15	0.32 ± 0.25	0.98
Plaque Length (mm)	0.60 ± 0.28	0.72 ± 0.29	0.76
Necrotic Core (%)	28.3 ± 10.20	24.3 ± 8.4	0.55
Macrophages (%)	40.0 ± 4.5	34.6 ± 13.1	0.79

## Discussion

Our study evaluated the effect of Ivabradine on heart rate and its subsequent effect on blood flow, WSS and plaque composition using a mouse model in which an advanced plaque was present. We demonstrated that Ivabradine significantly prolonged the duration of the diastolic phase, thus extending the entire cardiac cycle and reducing heart rate. Time averaged blood flow over the complete heart cycle did not change. Normalized peak blood flow was significantly higher in mice treated with Ivabradine, leading to a significant increase in normalized TAWSS during systole. These changes in WSS did not alter the plaque composition after 2 weeks of Ivabradine treatment.

Heart rate in mice treated with Ivabradine was 18.1% lower than the control mice, comparable to the values observed in other studies^15–20,26^. The control group showed an increase in heart rate. Such an increase was also reported by Drouin *et al*. in dyslipidaemic mice during aging^15^. This could be due to a combined effect of anesthesia and/or the use of contrast agent. To correct for this, we normalized the data to the baseline measurements. Ivabradine increased normalized peak blood flow in the RCCA to 124.2% ± 40.5% in our mice, which is comparable to the increase we measured in a single murine aorta previously^21^. In pigs of myocardial ischemia/reperfusion model, Heusch *et al*. reported a 67.5% increase in myocardial blood flow after the administration of Ivabradine^27^. In patients diagnosed with stable coronary artery disease, Ivabradine also induced an increase in flow i.e. a 12.4% increase in time-averaged peak coronary flow velocity during hyperemia. In our study, the increase in systolic blood flow did result in an increase in TAWSS during systole to 110.7% ± 18.2%, comparable to the increase in TAWSS we found previously in the murine aorta^21^. However, the TAWSS over the entire cardiac cycle was not affected by Ivabradine. Based on our current findings and the existing literature, Ivabradine treatment, regardless of the concentration or duration used, appeared to induce an increase in blood flow, but only during systole. This increase might be due to an Ivabradine-induced increase in stroke volume, as a prolonged diastolic phase is likely to result in an increase in left ventricle volume and thus a larger amount of blood leaving the left ventricle during systole. Due to the prolonged duration of diastole, the time-averaged blood flow during the complete cardiac cycle did not change in our study.

Ivabradine treatment for 6 weeks in LDLR^−/−^ mice resulted in a reduction in endothelial inflammation^21^, suggesting that although the increase in TAWSS is of short duration, it is sufficient to affect vascular biology. Pre-treatment with Ivabradine has been shown to prevent plaque growth in animal models of atherosclerosis in some^16,17^ but not all^20^ studies. Custodis *et al*. and Baumhäkel *et al*. observed that treatment of mice with Ivabradine significantly reduced plaque size in the aortic root and/or the ascending aorta^16,17^. However, it should be noted that both studies examined whether Ivabradine could prevent disease (i.e. they started Ivabradine treatment with the onset of high fat diet) but the potential effect of Ivabradine on plaque progression was not studied. We wished to know whether Ivabradine can be used to treat atherosclerosis and therefore analyzed whether Ivabradine treatment of an existing plaque would affect its composition. This hypothesis was supported by our previous report that changes in WSS over a developing plaque affects its phenotype^5^. However, we did not observe any changes in plaque composition in terms of plaque size, length, relative necrotic core area or relative macrophage accumulation in our current study. Our study design with 2 weeks of Ivabradine treatment did not show an effect on plaque size or composition. We set out, based on our power calculation, to analyse 5 control versus 5 Ivabradine-treated animals. However, due to the premature death of several animals, likely due to the combination of repeated anesthesia and the use of imaging contrast agent, we ended up with 4 control versus 5 Ivabradine-treated animals for histological analysis. Considering the P values we obtained (ranging from 0.55–0.98, see Table 3) we conclude there is no significant effect of Ivabradine on the plaque parameters we studied in our model. If we do a power calculation in hindsight, using the lowest p-value found, i.e. p = 0.55 for necrotic core 28.3 ± 10.20 versus 24.3 ± 8.4 (Table 3), we find we would need more than a 100 animals to reach statistical significance. Therefore, our findings point towards a lack of an effect of Ivabradine on plaque size and composition when it is administered after the initiation of disease. These data are in agreement with the clinical study by Fox *et al*. who did not observe a beneficial effect of Ivabradine on clinical outcome in patients who had coronary artery disease without heart failure^12^.

In conclusion, Ivabradine led to a small but significant increase in flow and WSS during the systolic phase of the heart cycle. Due to the prolonged diastolic phase, time-averaged flow and TAWSS over the complete heart cycle did not change. However, increased WSS during the systolic phase only accounts for a short duration of the cardiac cycle. This increase did not affect plaque size or composition in our animal model. Previous clinical and animal studies showed inconclusive results upon treatment with Ivabradine, with no or a positive effect.

## Electronic supplementary material

Supplemental information

## Data Availability

All data generated or analyzed during this study are included in this published article.
