# Supplementary material for: The effect of the heart rate lowering drug Ivabradine on hemodynamics in atherosclerotic mice
Source: Sci Rep. 2018 Sep 18;8:14014. doi: 10.1038/s41598-018-32458-3 (PMC6143553; doi:10.1038/s41598-018-32458-3)
Supplement: Supplementary file 1 — Supplemental information [file 41598_2018_32458_MOESM1_ESM.pdf]

Supplemental Material

The effect of the heart rate lowering drug Ivabradine on hemodynamics in atherosclerotic mice

R. Xing, A. M. Moerman, R.Y. Ridwan, K. van Gaalen, E.J. Meester, A.F.W. van der Steen, P. Evans, F. Gijsen, K. Van der Heiden

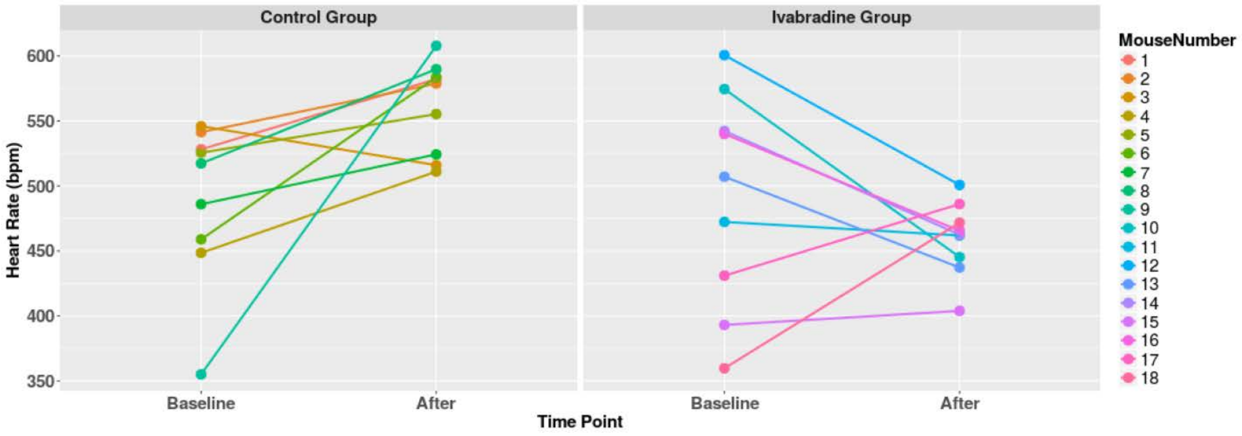

Supplemental Figure 1 Heart rate (beats per minute, bpm) for each individual mouse at baseline and 3 days after the onset of control (left) or Ivabradine (right) treatment.

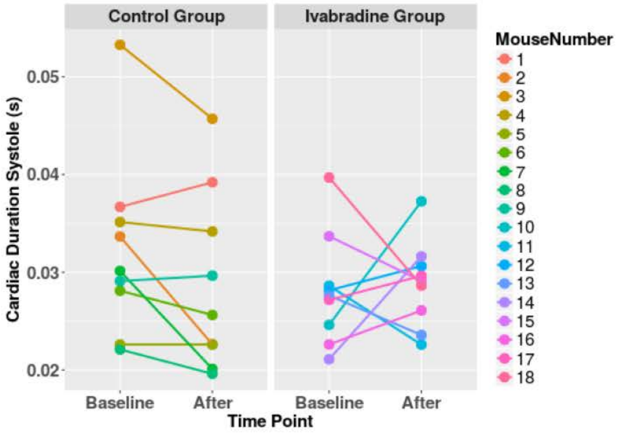

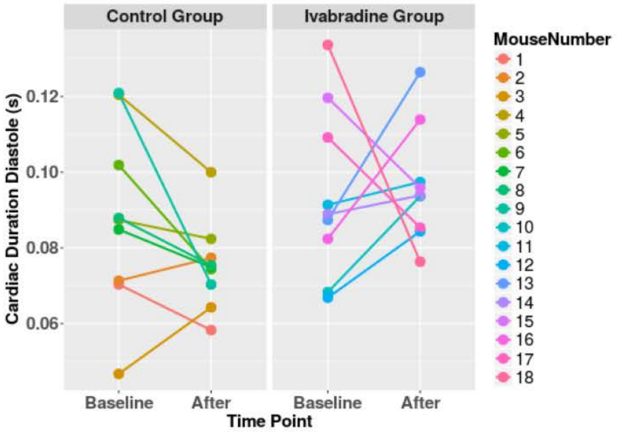

Supplemental Figure 2 Cardiac duration of systole (left) and diastole (right) for each individual mouse at baseline and 3 days after the onset of control (mouse number 1-9) or Ivabradine (mouse number 10-18) treatment.

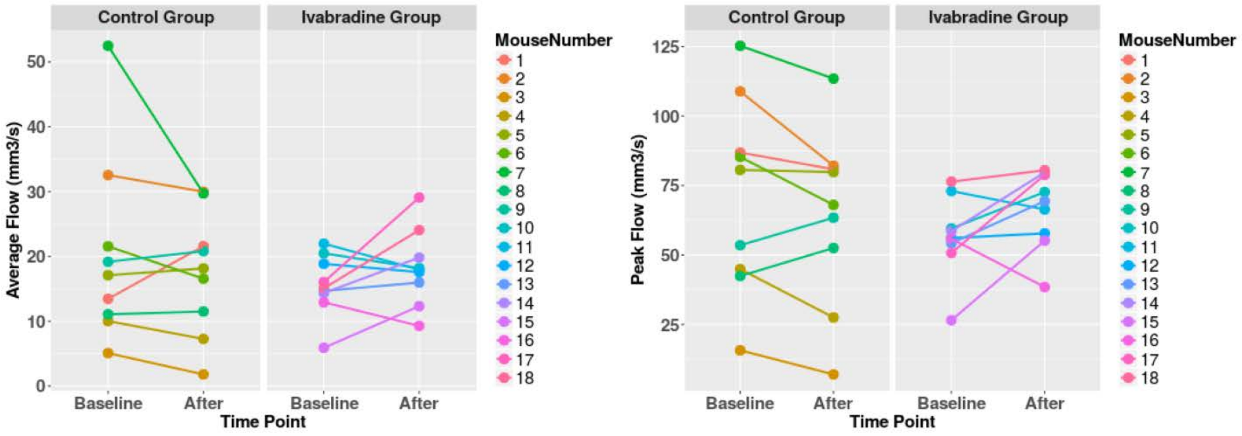

Supplemental Figure 3 Average (left) and peak (right) flow (mm3/s) for each individual mouse at baseline and 3 days after the onset of control (mouse number 1-9) or Ivabradine (mouse number 10-18) treatment.

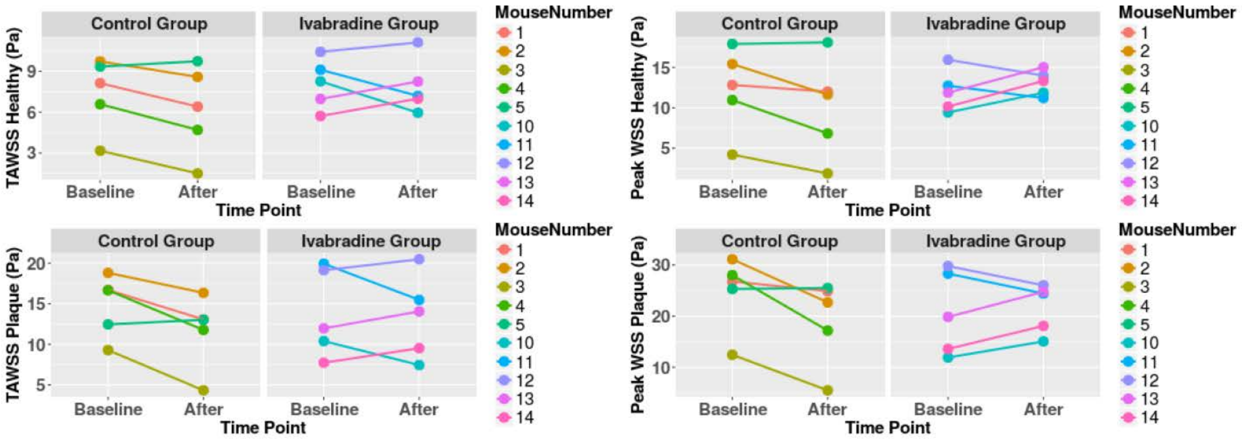

Supplemental Figure 4 Time averaged (left) and peak (right) WSS (Pa) for healthy (top) and plaque (bottom) segment for each individual mouse at baseline and 3 days after the onset of control (mouse number 1-5) or Ivabradine (mouse number 10-14) treatment.

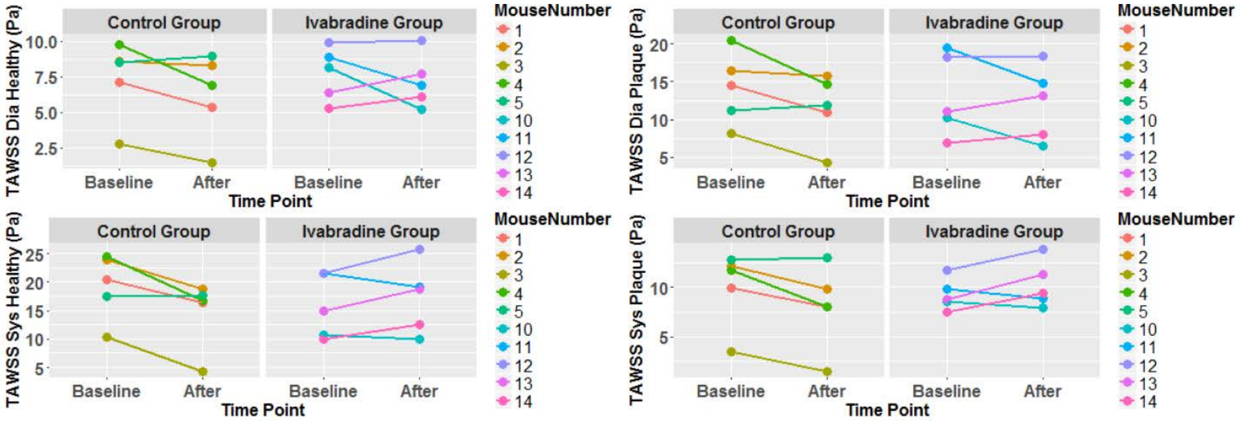

Supplemental Figure 5 Time averaged WSS (Pa) during diastole (top) and systole (bottom) for healthy (left) and plaque (right) segment for each individual mouse at baseline and 3 days after the onset of control (mouse number 1-5) or Ivabradine (mouse number 10-14) treatment.
